# Lymphadenopathy: Differentiation between Tuberculosis and Other Non-Tuberculosis Causes like Follicular Lymphoma

**DOI:** 10.3389/fpubh.2016.00031

**Published:** 2016-02-25

**Authors:** Karan Thakkar, Saket Mukund Ghaisas, Manmohan Singh

**Affiliations:** ^1^Pfizer, Mumbai, India

**Keywords:** CD20, acid fast bacilli, Koch’s disease, extrapulmonary tuberculosis, splenomegaly, lymphoma, lymphadenitis, GeneXpert

## Introduction

Lymphadenopathy (LD) is a common clinical presentation in outpatient departments (1–3). It is a cause of concern for the patient and physician alike even in the absence of symptoms (4). In India and some other developing countries, tuberculosis (TB) is the first differential diagnosis for a patient who presents with chronic lymph node enlargement (4–6). Nonetheless, studies have shown that more than 50% of cases of LD are due to non-TB causes and, in these cases, excision biopsy (EB) with histopathology and/or microbiological examination, is the only way to exclude TB (2, 5, 7). Malignancies may account for about 1% of cases of LD (1). The causes of LD can be broadly classified as shown below (8–10):

1. Malignancy: lymphoma, acute lymphoblastic leukemia, acute promyelocytic leukemia, and metastasis.
2. Infection: TB, streptococcal infection, pediculosis, measles, chickenpox, infectious mononucleosis, HIV, toxoplasmosis, and *Taenia*.
3. Autoimmune: systemic lupus erythematosus, serum sickness, juvenile idiopathic arthritis, and rheumatoid arthritis.
4. Drugs: phenytoin, cotrimoxazole, allopurinol, atenolol, penicillins, etc.
5. Miscellaneous: sarcoidosis, storage disorders, histiocytosis, cystic fibrosis, and hypothyroidism.

Of the various causes in adults, lymphoma and HIV infection should be ruled out in inconclusive cases (5). A retrospective analysis of 5 months data from a South African Lymphoma Clinic showed that out of 21 patients of lymphoma, 18 were mistakenly diagnosed as TB in the 12 months preceding the histological confirmation of lymphoma (11). Of the different types of lymphomas, the most common is “diffuse large B cell lymphoma” followed by “follicular lymphoma” (FL) (12, 13). FL is the most common indolent lymphoma (14). An early diagnosis of FL has become important in the light of availability of treatment options that can delay disease progression. LD due to FL and TB has a lot of similarities and differences. The article describes them with the objective of creating more awareness about FL enabling its early diagnosis. The presenting symptoms of FL are subtle, if any, and hence can be missed easily until disease progression to a more aggressive form (15–17).

## Lymphadenopathy: TB and FL – Similarities (14, 18)

1. Classically, TB LD has been described as multiple, matted, hard to fluctuant with draining sinuses. But in stages 1 and 2, lymph nodes may be discrete, firm, and rubbery just like FL LD (19).
2. Commonly involved lymph node regions in both (2, 7, 16, 17, 20, 21): cervical, axillary, and inguinal lymph nodes.
3. Both may have a non-specific presentation (9): fatigue, low grade fever, night sweats, weight loss, etc.
4. Fine needle aspiration cytology (FNAC): both TB and FL LD may show evidence of granulomatous inflammation (5).

## Lymphadenopathy: Clues Indicating Non-TB Etiology (14, 17, 18, 20, 22)

1. Age more than 40–50 years, since most cases of TB LD occur in 20–40 years age group.
2. Supraclavicular LD may point toward a malignancy (8).
3. No history of exposure to a case of TB.
4. An inconclusive repeat FNAC.
5. Normal tuberculin skin test (TST).
6. Lack of resolution of clinical symptoms and regression of LD after starting anti-TB therapy (ATT). In most cases of non-resistant TB LD, lymph node regression occurs within 2–4 months of start of ATT (23–25). Lack of response after 2 months of starting therapy should lead to consideration of resistance or non-TB causes.

**Figure 1:**
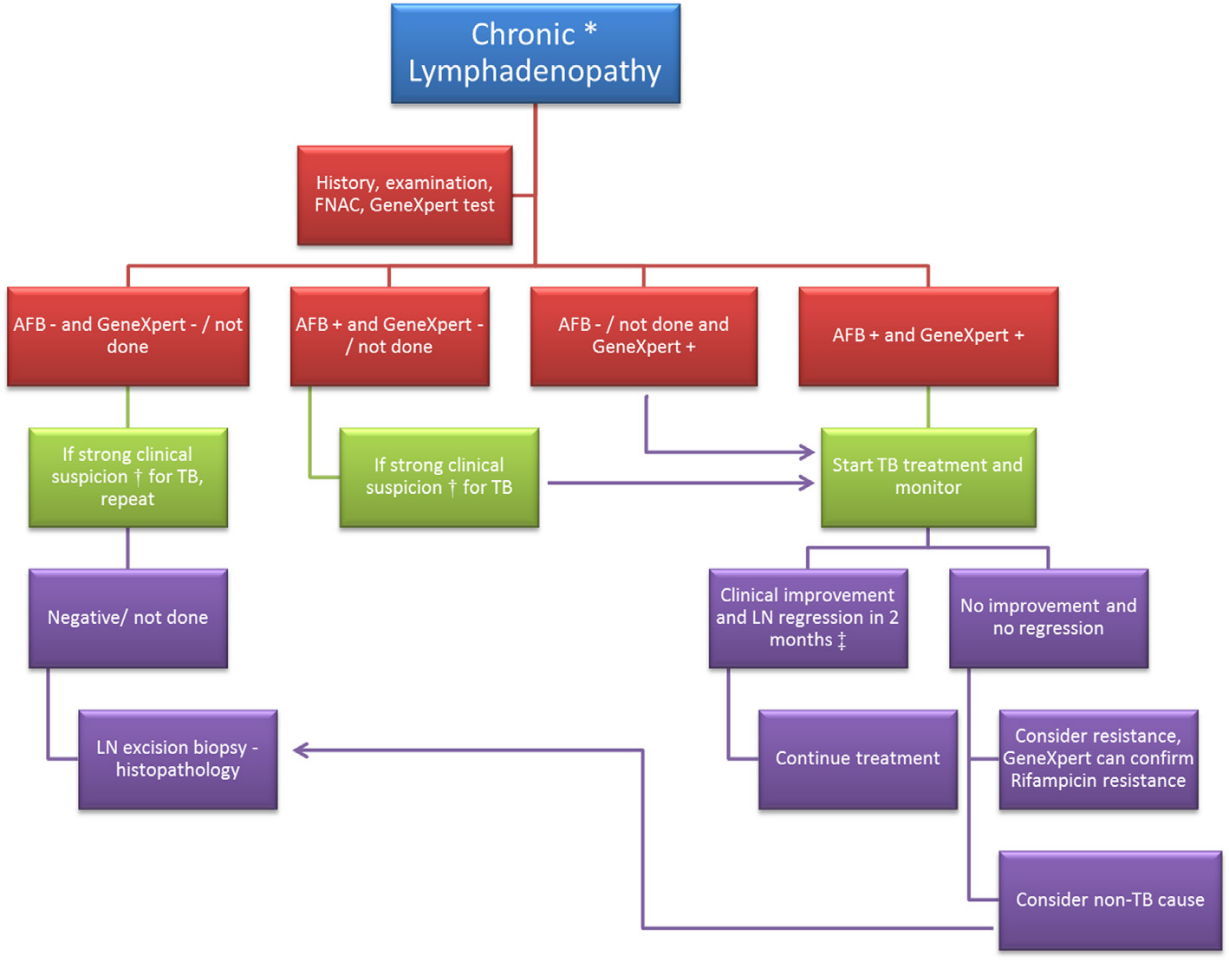
**Diagnostic algorithm in a case of chronic lymphadenopathy**. FNAC, fine needle aspiration cytology; AFB, acid fast bacilli; “−”, negative; “+”, positive; LN, lymph node. *Chronic lymphadenopathy: lymphadenopathy due to unexplained causes and duration ≥2 weeks. ^†^Clinical suspicion of tuberculosis (TB) would be based on (1) past history of TB or exposure to a case of TB; (2) age <50 years; (3) typical features of TB lymph node – multiple, matted, hard to fluctuant with draining sinuses; (4) symptoms like – fatigue, low grade fever, night sweats, weight loss, etc. ^‡^Based on studies conducted on response to treatment in cases of TB lymphadenopathy (23–25).

A general diagnostic algorithm in a case of chronic LD is presented in Figure 1.

## Chronic LD: Suggested Initial Workup

1. Complete blood counts, erythrocyte sedimentation rate, and a careful evaluation of the peripheral blood smear. (5, 15, 22, 26).
2. Tests such as serum lactate dehydrogenase, uric acid, and beta-2-microglobulin are good indicators of a lymphomatous process.
3. TST.
4. A chest x-ray may indicate mediastinal enlargement in case of FL or lesions of pulmonary TB.
5. Abdominal USG is an important non-invasive investigation in suspicious cases, since 50% of FL patients may have splenomegaly (26). Especially, if splenomegaly is massive, some of the differential diagnosis are chronic lymphocytic leukemia, non-Hodgkin lymphoma, chronic myelocytic leukemia, polycythemia vera, myelofibrosis with myeloid metaplasia, or hairy cell leukemia (27, 28). Splenic involvement in TB usually occurs in the military form and is very rare (29, 30).

Lymph node aspirate/biopsy for microscopy/histopathology and culture. Atypical lymphoid hyperplasia should be considered non-diagnostic rather than negative for a malignancy. Careful follow-up and an additional lymph node biopsy must be strongly considered in such cases (31). Fine needle aspiration (FNA) is an established investigation in the diagnosis of tuberculous lymphadenitis as it is a simple and relatively painless procedure (32–35). EB provides more information about histological architecture but requires surgical and anesthetic facilities and may leave a scar. The sensitivity of FNA and EB must be looked at in context of the combination diagnostic modality used. For example, for diagnosis of TB LAD, the sensitivity (95% CI) of FNA + cytological assessment is 38.5% (20–59%), FNA + microscopy for acid fast bacilli (AFB) is 18.2% (5–40%), and FNA + culture is 86.4% (65–97%), whereas that for EB + histological assessment is 95.8% (88–100%) and EB + microscopy for AFB is 16.7% (2–32%) (33). A study conducted by Singh et al. showed that EB was about twice as sensitive as FNA examination (*p* < 0.05) using the conventional diagnostic criteria of histology and culture for TB LAD (36). The same study also showed that, the positivity of polymerase chain reaction (PCR) in TB LAD cases using material from FNA and EB was same (10 out 22 cases of TB LAD) but by increasing the quantity of the clinical specimen for PCR analyses, more number of cases were diagnosed by EB (15/22) compared to FNA (12/22) (36). Thus, PCR on FNA samples can be performed as an initial test and EB histology and PCR should be recommended in cases negative on FNA + PCR, or if there is clinical suspicion for non-TB cases (36–38). For diagnosing lymphoma, although EB is an accepted “gold standard,” a core needle biopsy is also a viable alternative, provided that the number and size of cores for morphologic analysis are not compromised (39). GeneXpert test of lymph node biopsy material (40–42). It is a semiautomated and rapid method based on the detection of DNA of *Mycobacterium tuberculosis* and mutations responsible for rifampicin resistance (43). This method has been endorsed by the World Health Organization for rapid diagnosis of TB (44). Its utility is known for diagnosis of both pulmonary and extrapulmonary TB. According to a systematic review by Smith et al., GeneXpert test is likely to be of greatest utility when testing lymph node or tissue samples (besides CSF), and differentiating tuberculous from non-tuberculous mycobacteria in smear positive samples (43). A study has reported that the GeneXpert test reduced the time to begin ATT by 4 weeks in both pulmonary and extrapulmonary TB cases with a negative sputum AFB smear (45). The positive and negative predictive values of this test are 90–100 and 70–85%, respectively, since it has a specificity of almost 98–100% (42, 46–48). A negative test may not necessarily rule out TB but a positive test indicates TB; and hence, it has been described as a “rule-in” test. There are concerns about affordability but costs are being brought down followed by increased demand, support from health authorities, and public–private partnership (49, 50).

## Conclusion

Though FL is one of the less common causes of LD, there should be a high index of suspicion in selected cases to enable an early diagnosis. Key factors that would point toward a non-TB cause of LD are age >40 years, no history of TB, normal TST, inconclusive FNAC, negative GeneXpert test, and non-resolution of clinical symptoms and/or regression of LD in 2–4 months after starting ATT. An early diagnosis could permit the use of drugs, which have been shown to delay progression of FL.

## Author Contributions

KT, SG, and MS: contributed substantially to the conception of the work and interpretation of data for the work; involved in drafting the work and revising it critically for important intellectual content; approved the final version to be published; agreed to be accountable for all aspects of the work in ensuring that questions related to the accuracy or integrity of any part of the work are appropriately investigated and resolved.

## Conflict of Interest Statement

The authors are employees of Pfizer (India). Dr. KT is currently working as a Regional Medical Monitor on a Pfizer sponsored clinical trial on follicular lymphoma. Authors have prepared this article based on a literature review and the article does not include any official company stand/declaration or any Pfizer sponsored clinical trial data.
